# Rapid identification of *Lepiota brunneoincarnata* in China with loop-mediated isothermal amplification

**DOI:** 10.3389/fcimb.2026.1809128

**Published:** 2026-05-05

**Authors:** Danyang Fu, Yaya Sun, Jian Yu, Xiangrong Zheng, Jiajia Chen, Haijiao Li

**Affiliations:** 1 College of Landscape Architecture, Jiangsu Vocational College of Agriculture and Forestry, Zhenjiang, China; 2 Zhejiang Key Laboratory of Soil Remediation and Quality Improvement, Zhejiang A&F University, Hangzhou, China; 3 State Key Laboratory of Trauma and Chemical Poisoning, National Institute of Occupational Health and Poison Control, Chinese Center for Disease Control and Prevention, Beijing, China

**Keywords:** *Lepiota brunneoincarnata*, loop-mediated isothermal amplification, poisonous mushroom, *tef1*, visual identification

## Abstract

**Introduction:**

Many edible fungi and poisonous mushrooms share similar morphologic characteristics, making them difficult to distinguish with the naked eye. Lepiota brunneoincarnata is a major lethal toxic mushroom widely distributed in China and responsible for frequent poisoning incidents across the country.

**Methods:**

The present study developed a loop-mediated isothermal amplification (LAMP) reaction with a primer set designed from the translation elongation factor 1-a (tef1) sequence of L. Brunneoincarnata.

**Results:**

A set of specific LAMP primers were designed for the specific detection of L. brunneoincarnata, and the assay showed no amplification from 63 non-target fungal specimens. In this experiment, the limit of detection for DNA concentration was 100 pg/mL, and the limit of detection for spore concentration was 10 spores/mL. The method also successfully detected L. brunneoincarnata in boiled and digested mixed mushroom samples, and the whole detection procedure can be completed within 60 minutes at 63 °C.

**Discussion:**

The study is suitable for rapid identification of L. brunneoincarnata in China, and the method has potential for further optimization toward field application.

## 1 Introduction

Poisonous mushrooms (commonly referred to as toxic fungi or noxious mushrooms), defined as macrofungal fruiting bodies, have been reported to be associated with poisoning in humans or livestock upon ingestion (Li et al., 2022; Wijesekara and Xu, 2025). Most toxic species belong to Basidiomycetes, whereas a smaller proportion are classified within Ascomycetes (Bau et al., 2014; Zhang et al., 2019; Li et al., 2021). The macroscopic characteristics of poisonous mushrooms often closely resemble those of edible wild mushrooms, particularly when they co-exist in natural habitats. These morphologic similarities frequently lead to accidental foraging errors and subsequent poisoning (Jung et al., 2025). China continues to report annual incidents of mushroom poisoning, including fatal cases. Therefore, accurate identification of toxic species, characterization of toxin components, and classification of poisoning syndromes remain critical research priorities (Yuan et al., 2024). These investigations are essential for developing effective preventive strategies and advancing clinical treatment of mushroom intoxication. Current research data indicate that more than 14, 000 mushroom species have been identified to date worldwide. In China, scientific surveys have cataloged more than 3,800 species, including approximately 480 classified as poisonous (Wu et al., 2019). The genus *Lepiota* contains numerous poisonous mushrooms that are highly prone to accidental ingestion, leading to poisoning (Liu et al., 2018), including *L. brunneoincarnata*, *L. castanea*, and *L. venenata* (Ramirez et al., 1993; Ben et al., 2010; Naseer et al., 2022).

*Lepiota brunneoincarnata* is a deadly mushroom belonging to the genus *Lepiota* in the order Agaricales. It has a small white cap with a scale-like remnant that can split into concentric rings. Notably, it lacks a volva but possesses an annulus (ring) on the stem. This species is primarily distributed in Yunnan, Guizhou, Liaoning, and Jilin provinces of China. Despite its unremarkable appearance, this species contains both phallotoxins and amatoxins (Yilmaz et al., 2015). For most individuals, distinguishing the toxic mushroom *L. brunneoincarnata* from its edible counterparts poses a substantial challenge because of the striking resemblance in morphologic characteristics (Iturralde et al., 2010; Sun et al., 2019). From 2019 to 2021, *L. brunneoincarnata* was reported in 35 intoxication incidents in China, including 85 patient hospitalizations and 8 deaths (Jiang et al., 2023). In 2020, although *L. brunneoincarnata* was originally prevalent in Northeast, North, and Northwest China, it has recently expanded to Central, East, and Southwest China (Zhao et al., 2022). Therefore, establishing rapid and accurate identification methods is crucial for toxin source tracing and forensic confirmation in poisoning cases.

Current detection methods for *L. brunneoincarnata* include morphological analysis and DNA-based molecular identification. However, morphological approaches are inherently subjective and unreliable for distinguishing this species from closely related taxa (e.g., *L. cristata*) because of overlapping macroscopic characteristics. Morphological characteristics of fruiting bodies vary with developmental stages and environmental conditions (Li et al., 2024). Conventional polymerase chain reaction (PCR)–based molecular methods provide improved accuracy but require specialized instrumentation, extended processing time, and technical expertise, limiting their applicability in field settings and emergency scenarios. These constraints highlight the urgent need to develop rapid, simplified, efficient, and user-friendly detection methods that combine diagnostic accuracy with operational simplicity.

The development of loop-mediated isothermal amplification (LAMP), a nucleic acid amplification technique characterized by high efficiency, specificity, and rapidity, has provided a blueprint for the widespread adoption of rapid detection technologies. LAMP method uses 4 (to 6) specific primers and Bst DNA polymerase to exponentially amplify target sequences under isothermal conditions. Moreover, the addition of loop primers further accelerates the reaction process (Notomi et al., 2000). In recent years, LAMP has been widely used in fungal detection and identification (Kong et al., 2019), food safety inspection (Kumar, 2021), and environmental monitoring (Becherer et al., 2022). This technology demonstrates promising applications in genetic testing, particularly for rapid nucleic acid analysis under resource-limited conditions (Monika et al., 2019; Li et al., 2022). Furthermore, LAMP has been successfully applied for rapid detection of macrofungi, including *Chlorophyllum* (Wang et al., 2021), *Amanita* (Gao et al., 2022), *Russula* (Long et al., 2022), *Omphalotus* (Sugano et al., 2022), and *Gyromitra* (Xie et al., 2022). However, selecting the target genes is one of the critical factors for ensuring species-level specificity in LAMP detection. Currently, the primers designed for LAMP assays are primarily derived from the ITS region. Recent research has established an ITS-based LAMP detection system for *L. brunneoincarnata* through optimized primer design and reaction parameter standardization (Zhao et al., 2022). However, the limited number of genetic loci in ITS often fails to distinguish the closely related species. Consequently, identifying novel target genes has become a key focus of ongoing research.

In this study, we aimed to develop a rapid, sensitive, species-specific, and direct LAMP method for the detection of *L. brunneoincarnata* using the translation elongation factor 1-α (*tef1*) as the target gene.

## 2 Materials and methods

### 2.1 Sample collection

This study utilized 74 specimens of macrofungi which were maintained at the National Institute of Occupational Health and Poison Control, Chinese Center for Disease Control and Prevention (NIOHPCDC, **Table 1**).

**Table 1:** Samples for testing LAMP reaction specificity in this study.

Species	Sample no.	Geographic origin	Result
*Lepiota brunneoincarnata*	YNZT20210708-01	Yunnan	+
*L. brunneoincarnata*	XJKL20210809-01	Beijing	+
*L. brunneoincarnata*	Li20210915-11	Xingjiang	+
*L. brunneoincarnata*	HNLY20200607-01	Hunan	+
*L. brunneoincarnata*	GZ-BJ-DX-001	Ningxia	+
*L. brunneoincarnata*	SC20210815-11	Sichuan	+
*L. brunneoincarnata*	SD210904-17	Shandong	+
*L. brunneoincarnata*	LN20230822	Liaoning	+
*L. brunneoincarnata*	GZ20220815	Guizhou	+
*L. brunneoincarnata*	GD-SG-NX-02	Guangdong	+
*L. brunneoincarnata*	Chen 1898	Jiangsu	+
*L. acutesquamosa*	SD2020-05	Yunnan	–
*L. castanea*	Li171013-17	Qinghai	–
*L. castanea*	Chen 1895	Qinghai	–
*L. clypeolaria*	Chen 1892	Beijing	–
*L. clypeolaria*	Chen 1893	Beijing	–
*L. cristata*	Li20200729-12	Jiangsu	–
*L. cristata*	Chen 1894	Jiangsu	–
*L. lilacea*	Li 7843	Liaoning	–
*L. lilacea*	Li170906-11	Liaoning	–
*L. magnispora*	Li161015-62	Hebei	–
*L. subvenenata*	JS20200918-01	Jiangsu	–
*L. subvenenata*	Li170927-50	Jiangsu	–
*L. subvenenata*	JS20200918-01	Yunnan	–
*L. venenata*	Li171013-12	Hubei	–
*L. venenata*	Li171013-16	Hubei	–
*L. venenata*	Li171013-25	Hubei	–
*Amanita citrina*	Li150913-24	Guizhou	–
*A. sinocitrina*	GZHS20200703-04	Guizhou	–
*Chlorophyllum globosum*	ZH20190827-01	Guangdong	–
*C. hortense*	HNYZJH20210813-01	Hunan	–
*C. hortense*	GZRH20210819-01	Guizhou	–
*C. molybdites*	HZWX20210819-01	Zhejiang	–
*C. molybdites*	SCCD20210906-01	Sichuang	–
*C. sphaerosporum*	Li180726-02	Hubei	–
*C. sphaerosporum*	Li180728-13	Hubei	–
*Coniolepiota spongodes*	Li150830-53	Hubei	–
*Co. spongodes*	Li150830-54	Hubei	–
*Heinemannomyces splendidissimus*	Li180508-04	Hubei	–
*H. splendidissimus*	SZ20211016-03	Guangdong	–
*Lentinus edodes*	Chen1896	Zhejiang	–
*Le. edodes*	Chen1897	Zhejiang	–
*Leucoagaricus americanus*	ZJCA20210819-01	Zhejiang	–
*Leu. barssii*	Li20200822-25	Hubei	–
*Leu. leucothites*	NX20210917-47	Ningxia	–
*Leu. sinicus*	SZLH20211014-03	Guangdong	–
*Leu. vassiljevae*	Li170909-16	Hubei	–
*Leucocoprinus birnbaumii*	YBGAOX2021026	Jilin	–
*Leuc. cepistipes*	SZ20210420-02	Guangdong	–
*Leuc. cretaceus*	GDSZ20210606-01	Guangdong	–
*Leuc. cretaceus*	SZ20200527-01	Guangdong	–
*Leuc. lilacinogranulosus*	Li160715-01	Hubei	–
*Macrolepiota detersa*	HN20191031-01	Hunan	–
*M. subcitrophylla*	Li150915-39	Hubei	–
*Micropsalliota furfuracea*	HNHHZF20200915-01	Hunan	–
*Mi. globocystis*	Li170928-01	Hubei	–
*Mi. lateritia*	Li180704-01	Hubei	–
*Mi. megarubescens*	Li180508-05	Hubei	–
*Omphalotus yunnanensis*	YNHK20230717-01	Yunnan	–
*Pseudosperma arenarium*	NX20210922-57	Ningxia	–
*Russula aeruginea*	Li161014-25	Guizhou	–
*R. brevipes*	Li170608-03	Guizhou	–
*R. chloroides*	ML20170719-10	Yunnan	–
*R. compacta*	Li170927-43	Yunnan	–
*R. dinghuensis*	HNYZLL20210823-12	Hunan	–
*R. densifolia*	Li150830-39	Yunnan	–
*R. densifolia*	Li160728-05	Chongqing	–
*R. griseocarnosa*	ZJWZ20210624-02	Zhenjiang	–
*R. japonica*	ZJJH20201023-01	Zhenjiang	–
*R. leucocarpa*	SJWYS20210904-4	Fujian	–
*R. nigricans*	YNCX20210813-01	Yunnan	–
*R. rufobasalis*	HNLK20200612-10	Hunan	–
*R. subatropurpurea*	HNLK20200612-04	Hunan	–
*R. subnigricans*	SJWY20200730	Fujian	–

### 2.2 DNA extraction

All samples (11 samples of *L. brunneoincarnata* and 63 specimens of other non-*L. brunneoincarnata* fungi) were rapidly freeze-dried using liquid nitrogen and then thoroughly ground into fine powder. Genomic DNA was extracted from fruiting bodies or spore suspensions using the DNAsecure Plant Genomic DNA kit (TIANGEN Biotech, Beijing, China) according to the manufacturer’s instructions. After extraction, DNA concentration was quantified using a NanoDrop 2000 spectrophotometer (Thermo Fisher Scientific, USA), and the extracted genomic DNA concentration ranged from 80–150 ng/μL with A260/A280 ratios of 1.8–2.0 (indicating high purity), and the samples were stored in the TE buffer (10 mM Tris-HCl, 1 mM EDTA, pH 8.0) at −20 °C for subsequent use.

### 2.3 LAMP primers design

The *tef1* gene sequences of *L. brunneoincarnata* and *L. acutesquamosa* (GenBank accession numbers: PX965928, PX965929) have been deposited in GenBank (http://www.ncbi.nlm.nih.gov) for sequence alignment and species-specific region screening, which served as the basis for LAMP primer design. The *tef1* gene sequences of *L. brunneoincarnata* and its related species—*L. acutesquamosa*, *L. castanea*, *L. lilacea*, *L. magnispora*, *L. spiculata*, and *L. rhodophylla* (accession numbers: PX965929, MN820911, PP357892, MN820937, MK696577, and HM488950, respectively) were analyzed using BioEdit software (http://en.bio-soft.net/format/BioEdit.html; accessed on 20 February 2025). The alignment was to identify intraspecific conserved regions and interspecific variable regions within the *tef1* gene for species-specific primer design. Intraspecific conserved regions were defined as segments with ≥ 99% sequence homology and no insertions, deletions, or nucleotide mutations across all tested geographic populations of *L. brunneoincarnata*. Interspecific variable regions were defined as segments showing ≤ 85% sequence similarity and at least five consecutive species-specific nucleotide differences between *L. brunneoincarnata* and its closely related *Lepiota* species. Based on the alignment results, species-specific LAMP primers targeting the interspecific differentiated and intraspecific conserved regions of the *tef1* gene were designed using the online software Primer Explorer V5 (http://primerexplorer.jp/e; accessed on 25 February 2025). A total of twenty primer sets were created according to the principles of species-specific LAMP primer design, of which showed the greatest specificity were further screened using a broad range of species (**Table 2**).

**Table 2:** The species-specific LAMP primers for *Lepiota brunneoincarnata*.

Primer	Sequence (5′−3′)	Length	Target gene
F3 (forward outer)	CTCTTGGCCTTTACCCTTGG	20	*tef1*
B3 (backward outer)	CATGCCAGCCGGAAATGG	18	
FIP (forward inner) (F1c+F2)	CGGTGGAACTGAGGGATGGACA-TGTCCGTCAGCTCATCGT	40	
BIP (backward inner) (B1c+B2)	GGAGCGAGGACCGCTTCAAC-TCGGGTTGTAACCAACCTTC	40	
LF (loop forward)	TTGACAAACCTTGGTAGTGTCC	22	
LB (loop backward)	GAAATCGTCAGGGAAGTCACC	21	

### 2.4 LAMP reaction and analysis of specificity and suitability

The LAMP reaction system used in this study consisted of the following components: 2.5 μL of 10× ThermoPol buffer (New England BioLabs, Japan), 8 mmol/L MgSO_4_, 1.2 mmol/L dNTPs (TaKaRa Bio, China), 1.6 μmol/L each of FIP and BIP primers, 0.4 μmol/L each of F3 and B3 primers, 0.8 μmol/L each of LF and LB primers, 0.8 μmol/L betaine (Sigma-Aldrich, USA), 8 U/μL Bst DNA polymerase (New England BioLabs, Japan), 180 μmol/L hydroxynaphthol blue (HNB) (Aladdin, China), 2 μL of template DNA, and sterile water to a final volume of 26 μL. The mixtures for the LAMP reaction underwent heating at 60, 63, and 66°C for 60, 70, and 80min at each temperature to select the optimal temperature and the least time required for a successful reaction. Reaction results were determined by colorimetric analysis: a sky blue color indicated a positive result, whereas purple indicated a negative result (absence of *L. brunneoincarnata*). All experiments were performed in triplicate.

To assess the specificity of newly designed LAMP primers, genomic DNA from 11 specimens of *L. brunneoincarnata* and 63 samples of 47 other fungal species was subjected to LAMP amplification using the established reaction system mentioned above. All DNA samples were adjusted to 80–150 ng/μL. Non-target species served as negative controls to evaluate the specificity. Reaction specificity was determined by colorimetric analysis. Specificity was confirmed when positive control (*L. brunneoincarnata*) produced a sky blue color, whereas negative and blank controls remained purple. All experiments were performed in triplicate to ensure reliability.

### 2.5 LAMP sensitivity assay

The genomic DNA concentration of *L. brunneoincarnata* was measured using a microspectrophotometer (Thermo Fisher Scientific, USA) with 100–120 ng/µL, which was enough to meet the requirements of the subsequent study. Serial 10-fold dilutions of the DNA were prepared using sterile water to obtain 7 concentration gradients: 10 ng/μL, 1 ng/μL, 100 pg/μL, 10 pg/μL, 1 pg/μL, 100 fg/μL, and 10 fg/μL. For LAMP amplification, 2 μL of each diluted DNA sample was used as the template. Sterile water served as the negative control. Positive LAMP results were determined based on color change (Zhou et al., 2023). All experiments were performed in triplicate to ensure reliability. Time-to-threshold (Tt) was defined as the time required for a visible color change (from purple to sky blue) in the LAMP reaction at the limit of detection concentration. The coefficient of variation (CV) was calculated as the ratio of the standard deviation to the mean Tt value, expressed as a percentage, to evaluate the repeatability of the assay.

### 2.6 Spore concentration detection

**Figure 1:**
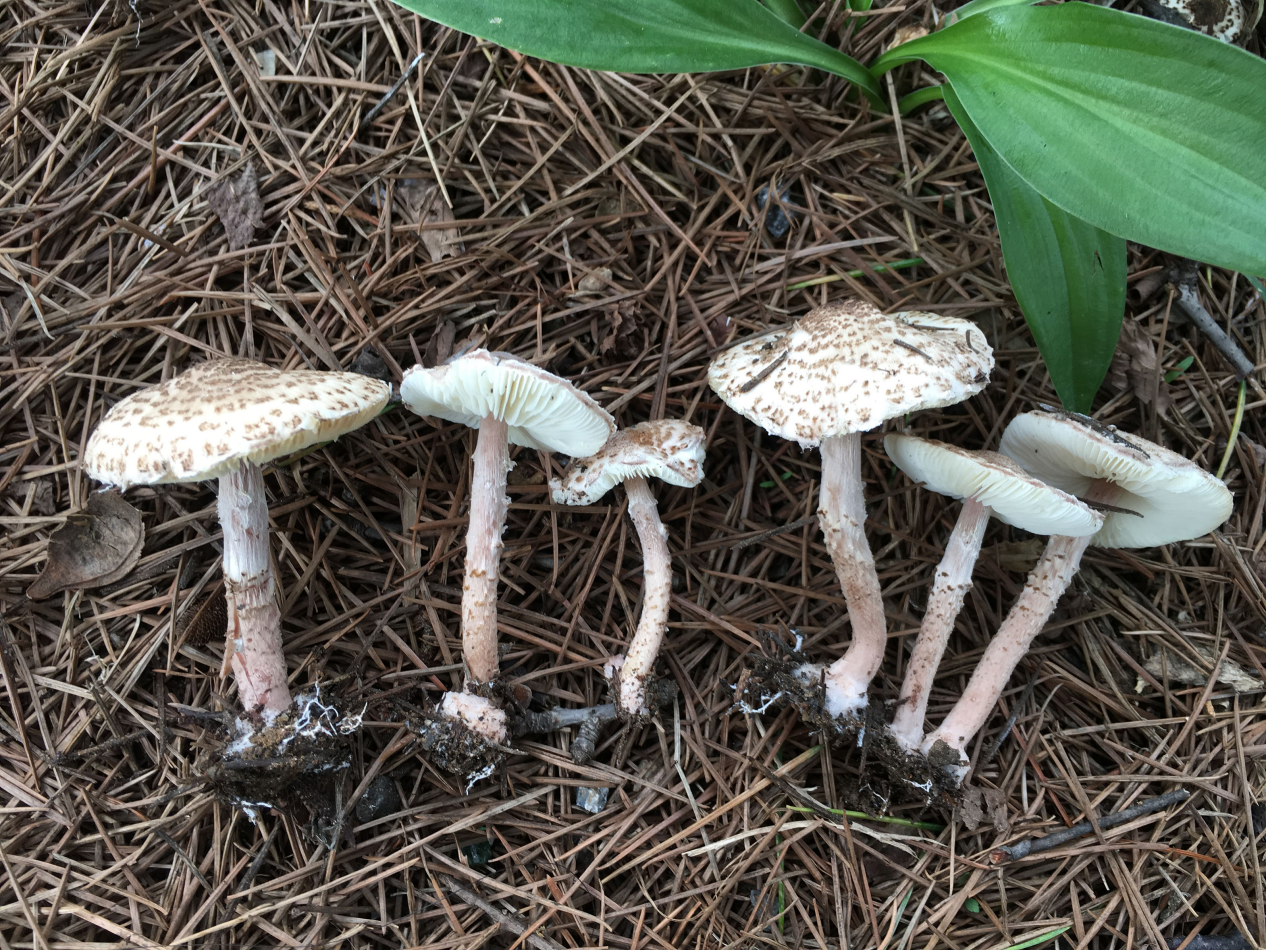
Basidiomata of *Lepiota brunneoincanata*.

Suspensions of basidiospores were obtained from *L. brunneoincarnata* (the representative sample, YNZT20210708-01) (**Figure 1**), with concentrations of 10^4^, 10^3^, 10^2^, 50, 10, and 0 spores per 1 μL. DNA from *L. brunneoincarnata* served as the positive control, and sterile water served as the negative control. All experiments were performed in triplicate to ensure reliability.

### 2.7 LAMP detection of heat-treated and digested *L. brunneoincarnata*

Because mushrooms are typically cooked before consumption, the effects of boiling and simulated gastric digestion on LAMP detection of *L. brunneoincarnata* were evaluated. To simulate real consumption scenarios, mixed mushroom samples were prepared by blending *L. brunneoincarnata* with the edible mushroom *Lentinula edodes* at mass ratios of 50:50, 10:90, and 1:99 (w/w). For boiling treatment, all mixed samples were heated in boiling water (100 °C) for 10 min and then cooled to room temperature. For simulated gastric digestion, the boiled mixed samples were incubated in simulated gastric fluid (prepared according to the Pharmacopoeia of the People’s Republic of China (2015 edition) with pepsin 32 mg/mL, pH 1.2) at 37 °C for 60 min with gentle shaking. DNA from *L. brunneoincarnata* served as the positive control, and sterile water was used as the negative control. .

## 3 Results

### 3.1 Development of the LAMP assay for *L. brunneoincarnata*

**Figure 2:**
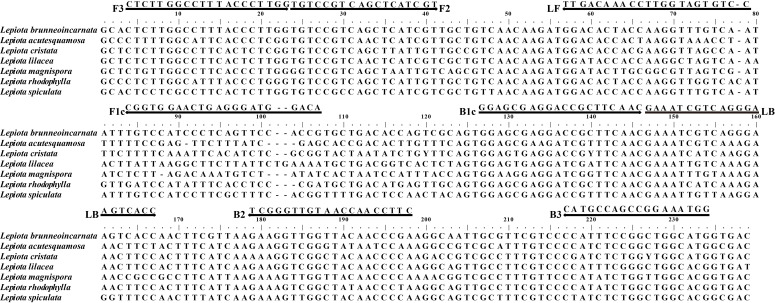
Nucleotide sequence alignment of the *tef1* region used for designing the LAMP primers. The primers used were F3, B3, FIP (F1c + F2), BIP (B1c + B2), LF, and LB.

Based on comparative analysis of *tef1* gene sequences from *L. brunneoincarnata* and closely related species, specific LAMP primers were designed, including outer primers (F3/B3), inner primers (FIP/BIP), and loop primers (LF/LB). The final primer set (**Table 2**) was chosen because it showed 100% specificity against 63 non-target fungi, the fastest and clearest color change, no non-specific amplification, and consistent performance in repeated tests. Other unsuccessful 19 primer pairs are listed in **Supplementary Table S1**. These primer sets enabled rapid and accurate detection of *L. brunneoincarnata* (**Figure 2**). The optimal reaction conditions were 63°C for 60 minutes under isothermal conditions (**Supplementary Figure S1**).

### 3.2 Specificity of the *tef1*-LAMP detection method

**Figure 3:**
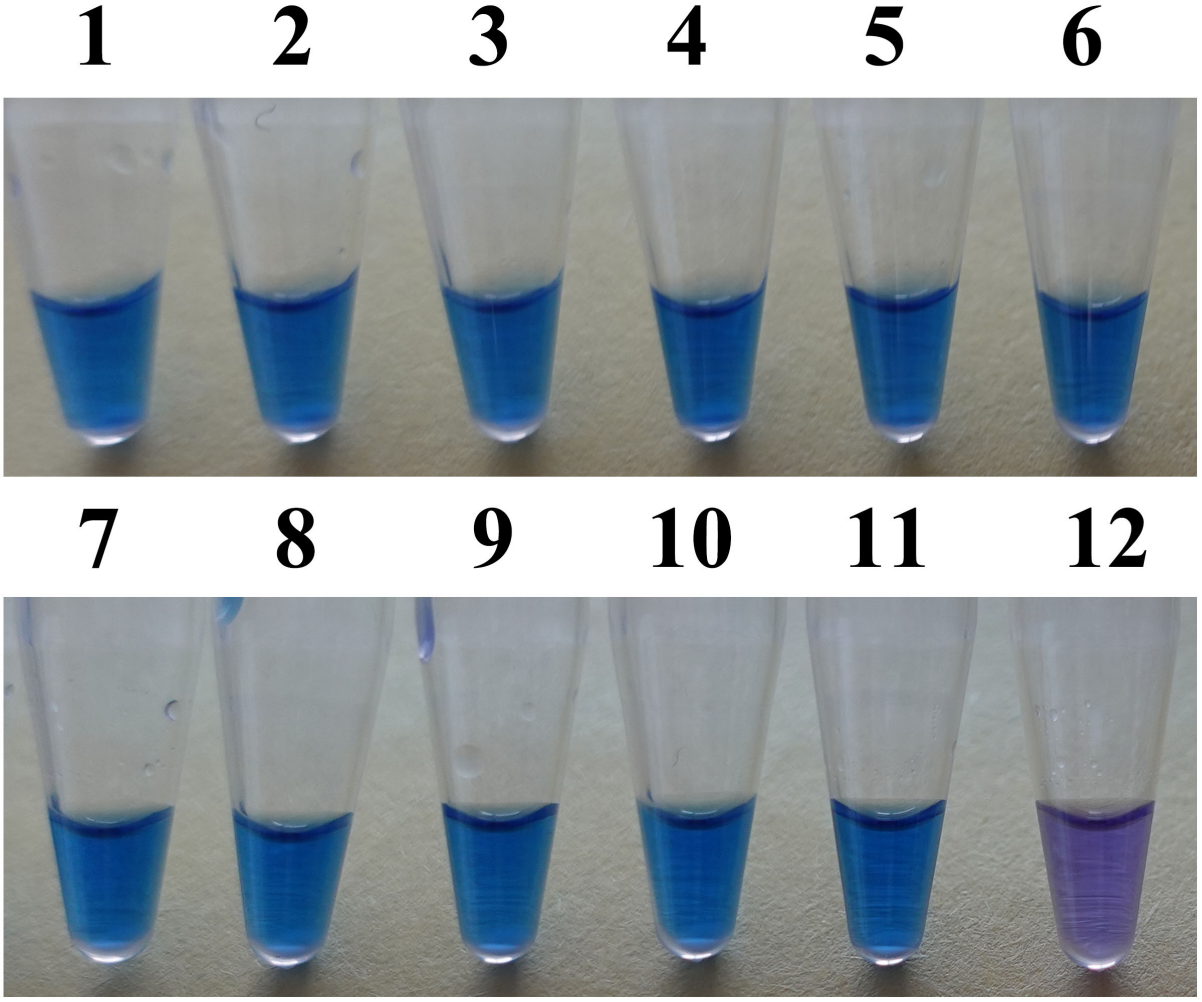
LAMP detection of the *tef1* gene in different isolates of *L. brunneoincarnata*. 1–11: *L. brunneoincarnata*; 12, Negative control.

**Figure 4:**
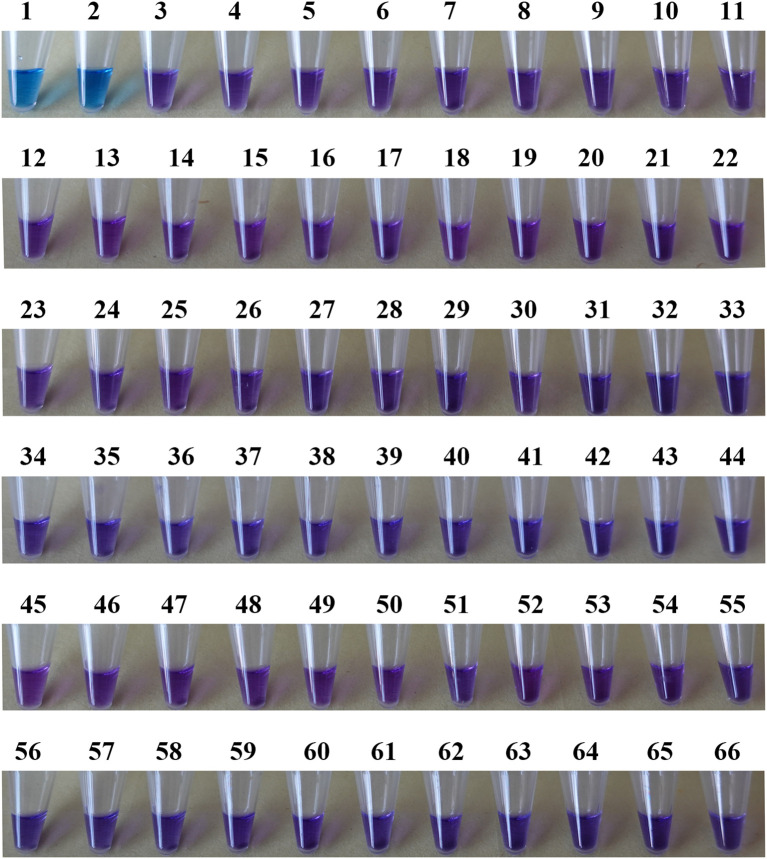
Specific detection of LAMP reactions in different DNA samples. 1–2, *Lepiota brunneoincarnata*; 3, *L. acutesquamosa*; 4–5, *L. castanea*; 6–7, *L. clypeolaria*; 8–9, *L. cristata*; 10–11, *L. lilacea*; 12, *L. magnispora*; 13–15, *L. subvenenata*; 16–18, *L. venenata*; 19, *Amanita citrina*; 20, *A. sinocitrina*; 21, *Chlorophyllum globosum*; 22–23, *C. hortense*; 24–25, *C. molybdites*; 26–27, *C. sphaerosporu*; 28–29, *Coniolepiota spongodes*; 30–31, *Heinemannomyces splendidissimus*; 32–33, *Lentinus edodes*; 34, *Leucoagaricus americanus*; 35, *Le.* barssii; 36, *Le. leucothites*; 37, *Le. sinicus*; 38, *Le. vassiljevae*; 39, *Leucocoprinus birnbaumii*; 40, *Leu. cepistipes*; 41–42, *Leu. cretaceus*; 43, *Leu. lilacinogranulosus*; 44, *Macrolepiota detersa*; 45, *M. subcitrophylla*; 46, *Micropsalliota furfuracea*; 47, *Mi. globocystis*; 48, *Mi. lateritia*; 49, *Mi. megarubescens*; 50, *Omphalotus yunnanensis*; 51, *Pseudosperma arenarium*; 52, *Russula aeruginea*; 53, *R. brevipes*; 54, *R. chloroides*; 55, *R. compacta*; 56, *R. dinghuensis*; 57–58, *R. densifolia*; 59, *R. griseocarnosa*; 60, *R. japonica*; 61, *R. leucocarpa*; 62, *R. nigricans*; 63, *R. rufobasalis*; 64, *R. subatropurpurea*; 65, *R. subnigricans*; 66, Negative control.

A reliable species-specific LAMP amplification primer set can demonstrate high interspecies specificity and strong intraspecies conservation. Using the optimized primer set (F3/B3, FIP/BIP, LF/LB) and optimized reaction conditions, the *tef1*–LAMP assay was evaluated using DNA from *L. brunneoincarnata* and 63 other fungal samples, with sterile water as the negative control. As shown in **Figures 3**, **4**, only the reaction tube containing *L. brunneoincarnata* DNA exhibited sky blue (positive result), whereas all other fungi and the negative control remained purple (negative result). These findings indicate high specificity of the *tef1*–LAMP primers for the detection of *L. brunneoincarnata*, including discrimination from closely related species.

### 3.3 Sensitivity of the *tef1*-LAMP detection method

**Figure 5:**
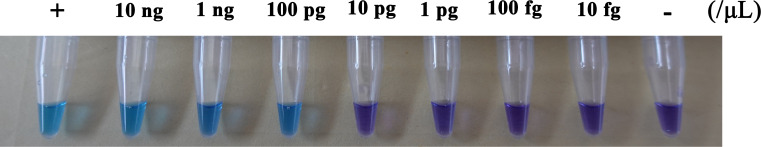
Sensitivity of LAMP assay using serially diluted genomic DNA of *L. brunneoincarnata*. +, Positive control; –, Negative control.

Sensitivity refers to the minimum detectable amount of DNA. The *tef1*–LAMP assay demonstrated a detection limit of 100 pg/μL for *L. brunneoincarnata* DNA, based on serial dilution from 10 ng/μL to 10 fg/μL (**Figure 5**). At the limit of detection concentration of 100 pg/μL, the mean Tt value was 58min, with a SD of 1min and a CV of 1.72%, demonstrating excellent repeatability and reliability of the *tef1*-LAMP assay.

### 3.4 Detection of spore suspensions

**Figure 6:**
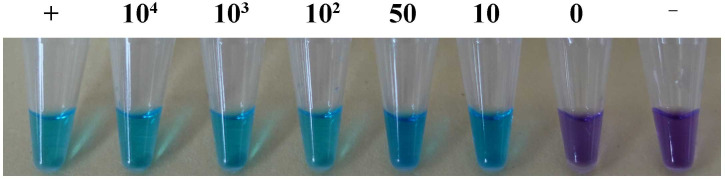
Efficiency of the LAMP assay in detecting *L. brunneoincarnata* containing different numbers of basidiospores. +, Positive control; –, Negative control.

In the LAMP assay, the positive control and spore suspensions at concentrations of 10^4^, 10^3^, 10^2^, 50, 10, and 0 basidiospores/μL exhibited a color change from purple to sky blue, whereas samples containing 0 basidiospores/μL and the negative control remained purple. These results indicate that the LAMP method can detect *L. brunneoincarnata* at a minimum concentration of 10 basidiospores/μL (**Figure 6**).

### 3.5 Detection in boiled and digested mushroom samples

**Figure 7:**
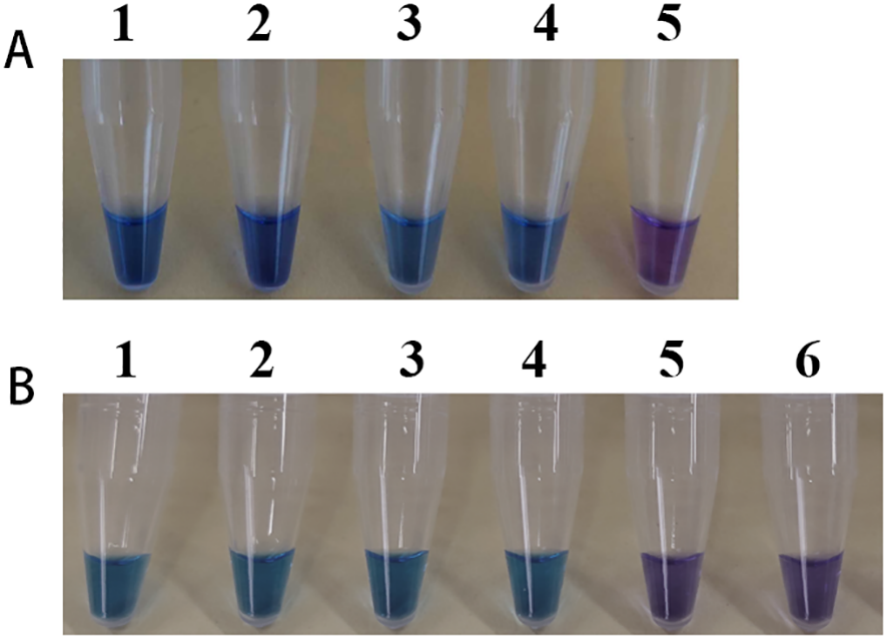
Suitability assessment of the LAMP reactions. Feasibility analysis of the LAMP method for on-site detection was evaluated by simulating mushroom processing and digestion. **(A)** LAMP assay used for the detection of boiled *L. brunneoincarnata*. 1, *L. brunneoincarnata*; 2, *L. brunneoincarnata*:*Lentinula edodes*= 50:50; 3, *L. brunneoincarnata*:*Lentinula edodes*= 10:90; 4, *L. brunneoincarnata*:*Lentinula edodes*= 1:99; 5, Negative control. **(B)** LAMP assay used for the detection of digested *L. brunneoincarnata* after boiled. 1, *L. brunneoincarnata*; 2, *L. brunneoincarnata*:*Lentinula edodes*= 50:50; 3, *L. brunneoincarnata*:*Lentinula edodes*= 10:90; 4, *L. brunneoincarnata*:*Lentinula edodes*= 1:99; 5, human saliva; 6: Negative control.

In practical applications, mushrooms are usually processed before consumption, and DNA degradation may occur during cooking and digestion. To evaluate the feasibility of the LAMP method under these conditions, mixed mushroom samples were prepared at different ratios and subjected to boiling and simulated gastric digestion. The results demonstrated that boiling and digestion did not compromise the ability of the LAMP assay to detect *L. brunneoincarnata* (**Figure 7**). These findings indicate that the LAMP method is suitable for detection of processed and digested *L. brunneoincarnata*, both in single-species samples and mixed mushroom preparations.

## 4 Discussion

Mushrooms are consumed worldwide as culinary staples; however, the ingestion of wild varieties poses a substantial risk of poisoning. This underscores the urgent need to develop reliable detection methods for accurate identification of toxic species. Rapid detection of mushrooms is essential for preventing poisoning and for timely identification of toxic species after ingestion (Wang et al., 2021; Gao et al., 2022). Current PCR amplification techniques are limited by lengthy sequencing times, high instrumentation requirements, and delayed post-analysis (Long et al., 2022). With advantages including operational simplicity, rapidity, high specificity, and cost-effectiveness, LAMP has emerged as a viable alternative to conventional PCR (Sugano et al., 2022). In this study, a LAMP assay targeting the *tef1* gene was developed for the rapid, specific, and visual detection of *L. brunneoincarnata*.

The ITS region is widely used for the identification of fungal species because of its moderate fragment length, high sequence polymorphism, and ability to reflect interspecies and intraspecies variation (Xu, 2016). ITS-based LAMP primers for *L. brunneoincarnata* have been successfully developed in previous studies (Jiang et al., 2023). However, reliance on ITS alone may be insufficient for discriminating closely related species. Phylogenetic analyses of *Lepiota* species based on 6 genetic markers (ITS, nrLSU, RPB1, RPB2, and *tef1*) have demonstrated that *tef1* sequences exhibit strong intraspecific conservation and significant interspecific divergence (Hou and Ge, 2020). The *tef1* is a highly conserved gene that encodes a eukaryotic translation elongation factor and contains both conserved and variable regions, making it a valuable marker for fungal phylogenetic studies. In genetic diversity analyses of *Ganoderma*, interspecific genetic differentiation values of *tef1* (0.012-0.038) were higher than those of ITS and nuclear large subunit (nLSU) markers, demonstrating superior resolution in distinguishing closely related species. In this study, multiple LAMP primer sets were designed by comparing *tef1* sequence variations between *L. brunneoincarnata* and closely related species. Through optimization of reaction conditions and incubation time, a specific *tef1*-LAMP detection method for *L. brunneoincarnata* was successfully established.

The core findings of this study demonstrate the robust performance of the established *tef1*-LAMP assay. The assay exhibited 100% specificity across 11 geographic isolates of *L. brunneoincarnata* and 63 non-target fungal samples, including closely related *Lepiota* species that are frequently misidentified by morphological traits. Regarding sensitivity, the assay achieved a detection limit of 100 pg/μL for genomic DNA and 10 spores/μL for spore suspensions, which is sufficient for practical detection in contaminated samples. Furthermore, the assay successfully detected *L. brunneoincarnata* in boiled and simulated gastric digested mushroom mixtures at ratios as low as 1: 99, confirming its applicability to processed and ingested samples—the most common scenario in clinical poisoning cases.

Reported LAMP assays for other fungal species sometimes showed a sensitivity of 100 fg/μL genomic DNA or shorter reaction time (30 min), while the detection limit of our assay was 100 pg/μL. The sensitivity was not improved by adjusting reaction temperature or duration, which might be probably attributed to the copy number of the *tef1* target or the reaction components.

In conclusion, the developed LAMP method demonstrates high specificity, sensitivity, and rapidity for the detection of *L. brunneoincarnata* in China. This technology holds significant potential for ensuring future food quality and safety.

## Data Availability

The datasets presented in this study can be found in online repositories. The names of the repository/repositories and accession number(s) can be found in the article/**Supplementary Material**.

## Supplementary material

Supplementary Figure S1 The LAMP assay at different temperatures and times. 1, 60min; 2, 70min; 3, 80min; -, Negative control.

Supplementary Table S1 The unsuccessful species-specific LAMP primers for *Lepiota brunneoincarnata*.
