# Supplementary figures and images for: Rapid identification of Lepiota brunneoincarnata in China with loop-mediated isothermal amplification
Source: Front Cell Infect Microbiol. 2026 May 5;16:1809128. doi: 10.3389/fcimb.2026.1809128 (PMC13183844; doi:10.3389/fcimb.2026.1809128)

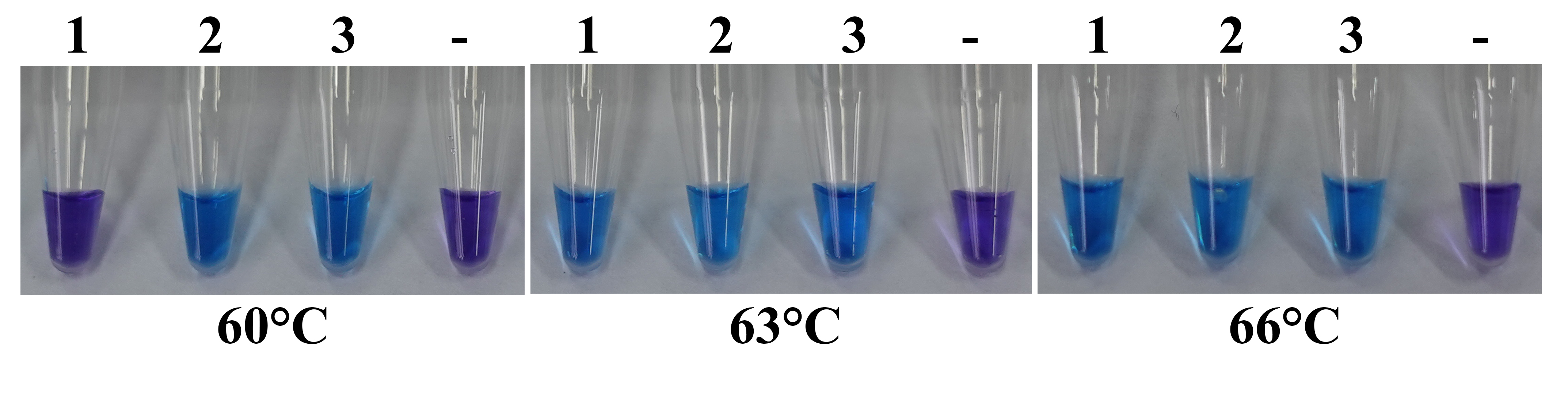

Supplement: Supplementary Figure S1 — The LAMP assay at different temperatures and times. 1, 60min; 2, 70min; 3, 80min; -, Negative control. [file Image1.jpeg]
